# Characterization of Typhoid Intestinal Perforation in Africa: Results From the Severe Typhoid Fever Surveillance in Africa Program

**DOI:** 10.1093/ofid/ofad138

**Published:** 2023-06-02

**Authors:** Megan Birkhold, Shrimati Datta, Gi Deok Pak, Justin Im, Olakayode O Ogundoyin, Dare I Olulana, Taiwo A Lawal, Oludolapo O Afuwape, Aderemi Kehinde, Marie-France Phoba, Gaëlle Nkoji, Abraham Aseffa, Mekonnen Teferi, Biruk Yeshitela, Oluwafemi Popoola, Michael Owusu, Lady Rosny Wandji Nana, Enoch G Cakpo, Moussa Ouedraogo, Edgar Ouangre, Isso Ouedraogo, Anne-Sophie Heroes, Jan Jacobs, Ondari D Mogeni, Andrea Haselbeck, Leah Sukri, Kathleen M Neuzil, Octavie Lunguya Metila, Ellis Owusu-Dabo, Yaw Adu-Sarkodie, Abdramane Soura Bassiahi, Raphaël Rakotozandrindrainy, Iruka N Okeke, Raphaël M Zellweger, Florian Marks

**Affiliations:** Center for Vaccine Development and Global Health, University of Maryland School of Medicine, Baltimore, Maryland, USA; Center for Vaccine Development and Global Health, University of Maryland School of Medicine, Baltimore, Maryland, USA; Epidemiology, Public Health, and Impact Unit (EPIC), International Vaccine Institute, Seoul, Republic of Korea; Epidemiology, Public Health, and Impact Unit (EPIC), International Vaccine Institute, Seoul, Republic of Korea; Division of Pediatric Surgery, University College Hospital and Department of Surgery, University of Ibadan, Ibadan, Nigeria; Division of Pediatric Surgery, University College Hospital and Department of Surgery, University of Ibadan, Ibadan, Nigeria; Division of Pediatric Surgery, University College Hospital and Department of Surgery, University of Ibadan, Ibadan, Nigeria; Division of Gastrointestinal Surgery, University College Hospital and Department of Surgery, University of Ibadan, Ibadan, Nigeria; Department of Medical Microbiology and Parasitology, College of Medicine, University of Ibadan, Ibadan, Nigeria; Department of Microbiology, Institut National de Recherche Biomedicales, Kinshasa, Democratic Republic of Congo; Department of Microbiology, Institut National de Recherche Biomedicales, Kinshasa, Democratic Republic of Congo; Armauer Hansen Research Institute, ALERT Campus, Addis Ababa, Ethiopia; Armauer Hansen Research Institute, ALERT Campus, Addis Ababa, Ethiopia; Armauer Hansen Research Institute, ALERT Campus, Addis Ababa, Ethiopia; Department of Community Medicine, College of Medicine, University of Ibadan, Ibadan, Nigeria; Department of Medical Diagnostics, Kwame Nkrumah University of Science and Technology, Kumasi, Ghana; Institut Supérieur des Sciences de la Population, Université Joseph Ki Zerbo, Ouagadougou, Burkina Faso; Institut Supérieur des Sciences de la Population, Université Joseph Ki Zerbo, Ouagadougou, Burkina Faso; Laboratorie d'Analyses Medicales, Hopital Protestant Schiphra, Ouagadougou, Burkina Faso; Service de Chirurgie Viscérale, Hopital Yalgado, Ouagadougou, Burkina Faso; Pediatric Department, Hopital Charles de Gaulle, Ouagadougou, Burkina Faso; Department of Tropical Bacteriology, Institute of Tropical Medicine, Antwerp, Belgium; Department of Microbiology, Immunology and Transplantation, KU Leuven, Leuven, Belgium; Department of Tropical Bacteriology, Institute of Tropical Medicine, Antwerp, Belgium; Department of Microbiology, Immunology and Transplantation, KU Leuven, Leuven, Belgium; Epidemiology, Public Health, and Impact Unit (EPIC), International Vaccine Institute, Seoul, Republic of Korea; Epidemiology, Public Health, and Impact Unit (EPIC), International Vaccine Institute, Seoul, Republic of Korea; Center for Vaccine Development and Global Health, University of Maryland School of Medicine, Baltimore, Maryland, USA; Center for Vaccine Development and Global Health, University of Maryland School of Medicine, Baltimore, Maryland, USA; Department of Microbiology, Institut National de Recherche Biomedicales, Kinshasa, Democratic Republic of Congo; Service de Microbiologie, Cliniques Universitaires de Kinshasa, Kinshasa, Democratic Republic of Congo; Department of Medical Diagnostics, Kwame Nkrumah University of Science and Technology, Kumasi, Ghana; Department of Medical Diagnostics, Kwame Nkrumah University of Science and Technology, Kumasi, Ghana; Institut Supérieur des Sciences de la Population, Université Joseph Ki Zerbo, Ouagadougou, Burkina Faso; Department of Microbiology and Parasitology, University of Antananarivo, Antananarivo, Madagascar; Faculty of Pharmacy, University of Ibadan, Ibadan, Nigeria; Epidemiology, Public Health, and Impact Unit (EPIC), International Vaccine Institute, Seoul, Republic of Korea; Epidemiology, Public Health, and Impact Unit (EPIC), International Vaccine Institute, Seoul, Republic of Korea; Cambridge Institute of Therapeutic Immunology and Infectious Disease, University of Cambridge School of Clinical Medicine, Cambridge Biomedical Campus, Cambridge, United Kingdom; Heidelberg Institute of Global Health, University of Heidelberg, Heidelberg, Germany; Madagascar Institute for Vaccine Research, University of Antananarivo, Antananarivo, Madagascar

**Keywords:** *Salmonella* Typhi, intestinal perforation, Africa, typhoid fever, severe typhoid

## Abstract

**Background:**

Typhoid intestinal perforation (TIP) remains the most serious complication of typhoid fever. In many countries, the diagnosis of TIP relies on intraoperative identification, as blood culture and pathology capacity remain limited. As a result, many cases of TIP may not be reported as typhoid. This study demonstrates the burden of TIP in sites in Burkina Faso, Democratic Republic of Congo (DRC), Ethiopia, Ghana, Madagascar, and Nigeria.

**Methods:**

Patients with clinical suspicion of nontraumatic intestinal perforation were enrolled and demographic details, clinical findings, surgical records, blood cultures, tissue biopsies, and peritoneal fluid were collected. Participants were then classified as having confirmed TIP, probable TIP, possible TIP, or clinical intestinal perforation based on surgical descriptions and cultures.

**Results:**

A total of 608 participants were investigated for nontraumatic intestinal perforation; 214 (35%) participants had surgically-confirmed TIP and 33 participants (5%) had culture-confirmed typhoid. The overall proportion of blood or surgical site *Salmonella enterica* subspecies *enterica* serovar Typhi positivity in surgically verified TIP cases was 10.3%. TIP was high in children aged 5–14 years in DRC, Ghana, and Nigeria. We provide evidence for correlation between monthly case counts of *S.* Typhi and the occurrence of intestinal perforation.

**Conclusions:**

Low *S.* Typhi culture positivity rates, as well as a lack of blood and tissue culture capability in many regions where typhoid remains endemic, significantly underestimate the true burden of typhoid fever. The occurrence of TIP may indicate underlying typhoid burden, particularly in countries with limited culture capability.

Ingestion of food or water contaminated with the bacterium *Salmonella enterica* subspecies *enterica* serovar Typhi (*S.* Typhi) causes typhoid fever. Due to its similarity to other common gastrointestinal and febrile illnesses, typhoid fever can often be difficult to diagnose. Delays in diagnosis and treatment can lead to serious complications, including gastrointestinal hemorrhage and typhoid intestinal perforation (TIP) [1]. Improvements in water quality, food-handling practices, sanitation infrastructure, and access to antibiotics have contributed to dramatic declines in the incidence and mortality of typhoid fever in high-income countries [2, 3]. However, there are still >9 million cases of typhoid fever and >110 000 attributable deaths globally each year, with a disproportionate amount of these cases occurring in low- and middle-income countries [4].

TIP is a life-threatening complication, which requires emergency surgery and can lead to increased morbidity and mortality. In sub-Saharan Africa, perforation-associated mortality rates range from 10% to 30% [5]. TIP disproportionately affects children and marginalized populations without access to care. In 1 rural Nigerian hospital, almost 50% of surgical acute abdomens were attributed to typhoid perforation, and 72% of TIP cases occurred in children [6].

TIP is often confirmed intraoperatively with the typical presentation being an oval perforation along the antimesenteric border of the terminal ileum, although the location of perforation has also been reported in both the jejunum and colon, and, less commonly, the gallbladder [7–10]. Unfortunately, in many of the areas where typhoid occurs, microbiology and pathology capacity are also limited. Therefore, these intestinal perforations (IPs) may not be reported as typhoid, and the burden of typhoid is underestimated. Accurate ascertainment of typhoid burden is particularly relevant, given the availability of World Health Organization (WHO)–recommended, affordable typhoid conjugate vaccines (TCVs) that are suitable for use in campaigns and routine pediatric immunization programs [1].

While data are limited, case reports and case series suggest that the burden of TIP in sub-Saharan Africa is highly variable and may be substantial in certain settings [5, 11, 12]. The Severe Typhoid in Africa Program (SETA), conducted in 6 countries, investigated bacterial etiologies of fever, including severe complications [13]. Using the SETA data, our goal was to describe the epidemiology, clinical profiles, and associated risk factors for nontraumatic IPs in children and adults, and to categorize these cases based on their likelihood of being TIP. Furthermore, as blood cultures were routinely obtained as part of SETA, we sought to model the association between IPs and blood culture–confirmed typhoid fever in these countries [14].

## METHODS

This study included participants enrolled in the SETA program in 6 countries (Burkina Faso, Democratic Republic of Congo [DRC], Ethiopia, Ghana, Madagascar, and Nigeria); detailed methods of the study have been previously published [13]. Patients of all ages residing in the defined catchment areas within each of the participating countries meeting fever criteria (defined as having a temperature of ≥37.5°C axillary or ≥38.0°C tympanic measurement upon examination or a history of 3 consecutive days of self-reported fever within the preceding 7 days) were eligible for inclusion in the surveillance study and were evaluated for bacteremia by blood culture diagnostics. Patients with clinical suspicion of nontraumatic peritonitis, as determined by the study physician, were enrolled in the study regardless of fever status, and blood cultures were then collected. When surgery was performed on enrolled patients, a tissue biopsy from the edge of the perforation and a sample of peritoneal fluid were collected for culture. Cultures of blood and peritoneal fluid were conducted using automated systems (BACTEC Peds Plus Medium/BACTEC Plus Aerobic-F, BACTEC, Becton-Dickinson, Franklin Lakes, New Jersey or BacT/ALERT PF Pediatric FAN/BacT/ALERT FA FAN Aerobic, bioMérieux, Marcy l’Etoile, France) except for in DRC where specimens were inoculated in BacTAlert bottles, incubated at 37°C, and monitored by visual inspection. Tissue specimens were first ground using a mortar and pestle or a tissue grinder together with a small amount of sterile saline. Ground specimens were then plated on blood agar, MacConkey III agar, XLD Agar/SS agar, and enrichment medium (eg, thioglycolate broth). Blood agar was incubated at 5% carbon dioxide and other media were incubated in ambient conditions at 37°C for 18–24 hours. Further details on microbiological confirmation of *S.* Typhi have been published previously [13].

We classified patients into groups based on microbiological and surgical confirmation, as follows:

- Confirmed TIP: Intestinal perforation cases confirmed during surgery AND positive blood or surgical site culture for *S.* Typhi
- Probable TIP: Intestinal perforation cases confirmed during surgery AND no blood or surgical site culture confirmation for *S.* Typhi (ie, negative or not done)
- Possible TIP: Intestinal perforation cases without surgical confirmation of typhoid or no surgical records available AND positive blood or surgical site cultures for *S.* Typhi
- Clinical IP: Intestinal perforation cases without surgical confirmation of typhoid or no surgical records available AND no blood or surgical site culture confirmation for *S.* Typhi (ie, negative or not done)

Surgical specimens used for culture included peritoneal fluid and tissue specimens. Confirmation of TIP during surgery was obtained from surgical procedure notes, which either stated the perforation was due to typhoid or used descriptions consistent with a typical typhoid lesion—oval perforation along the antimesenteric border of the intestine.

Demographic, clinical, outcomes, and other related data were captured using Microsoft FoxPro version 9.0 (Microsoft, Redmond, Washington) and SETACOLLECT (Android version 5.0.1) during the study period.

Frequency and distribution of cases by country, stratification by age group and sex for each case definition, and summary statistics of clinical signs and symptoms for each case definition were analyzed using SAS software version 9.4 (SAS Institute, Cary, North Carolina). Graphs for number of cases by age group and sex and percentage of cases of the recruited population were made using GraphPad Prism version 8.4.2 software.

For the time series analysis, monthly counts of perforation and blood culture–confirmed *S.* Typhi were analyzed for the study period in aggregate and by country. The generalized linear model (GLM) analysis was implemented for estimating perforations attributed to *S.* Typhi. All analyses were implemented using SAS software version 9.4.

## RESULTS

**Figure 1.**
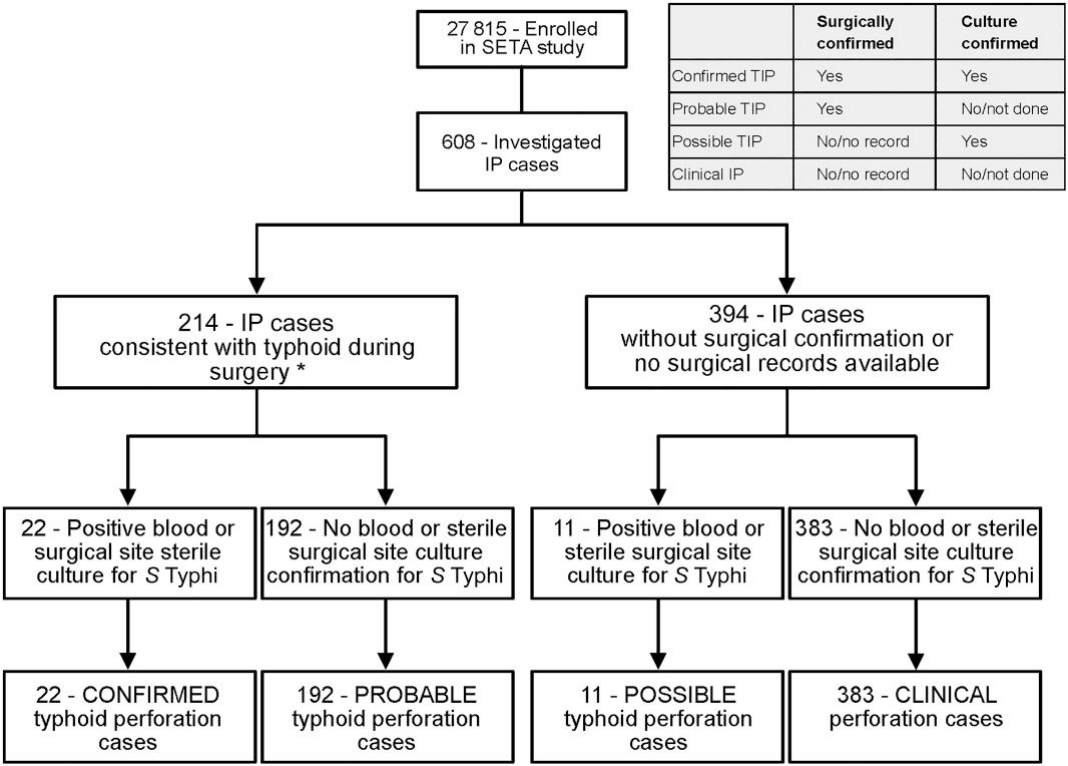
Study schematic and summary classification of typhoid intestinal perforations (inset). *Intestinal perforation confirmed during surgery or surgical evidence of a perforation or description including typhoid perforation, ileal perforation, and/or antimesenteric. Abbreviations: IP, intestinal perforation; SETA, Severe Typhoid in Africa Program; TIP, typhoid intestinal perforation.

Of 27 815 subjects enrolled in SETA, 608 patients underwent abdominal surgery and were investigated for TIP (Figure 1). In aggregate, TIP was confirmed through surgery in 214 individuals (214/608 [35%]). Overall, typhoid was culture confirmed in 33 surgical patients (33/608 [5%]), of which 22 were also surgically-confirmed (confirmed TIP) and 11 were not surgically-confirmed, mostly due to lack of availability of surgical records (possible TIP).

Overall, the rate of blood or surgical site culture *S.* Typhi positivity for surgically verified TIP was 10% (22/214). In a majority of surgical perforations (192/214 [90%]), diagnosis was not confirmed by microbiology despite surgical notes indicating that (1) perforation was due to typhoid or (2) perforation contained a description consistent with a typical typhoid lesion (Figure 1). We identified 383 (383/608 [63%]) surgical cases that were not confirmed by surgery or culture (clinical IP) (Figure 1).

When evaluated by country, DRC and Nigeria had the largest absolute number of investigated cases (198 and 173, respectively; Table 1). Once normalized to the entire population recruited in the study, Nigeria had the highest percentage of investigated TIPs (173/4356 [4%]), followed by Ghana (73/2182 [3%]), DRC (198/6239 [3%]), Burkina Faso (112/6199 [2%]), Ethiopia (41/5366 [1%]), and Madagascar (11/3473 [<1%]) (Supplementary Table 1).

**Table 1.** Number of Cases by Country

Case Definition	Country					
	Burkina Faso	DRC	Ethiopia	Ghana	Madagascar	Nigeria
Confirmed TIP (n = 22)	0 (0)	14 (7)	1 (2)	6 (8)	0 (0)	1 (1)
Probable TIP (n = 192)	26 (23)	90 (45)	8 (20)	40 (55)	2 (18)	26 (15)
Possible TIP (n = 11)	0 (0)	4 (2)	0 (0)	3 (4)	0 (0)	4 (2)
Clinical IP (n = 383)	86 (77)	90 (46)	32 (78)	24 (33)	9 (82)	142 (82)
Total (N = 608)	112 (100)	198 (100)	41 (100)	73 (100)	11 (100)	173 (100)

Data are presented as No. (column %).

Abbreviations: DRC, Democratic Republic of Congo; IP, intestinal perforation; TIP, typhoid intestinal perforation.

Of all 22 confirmed TIP cases, the majority were identified in DRC (14/22 [64%]), followed by Ghana (6/22 [27%]). There were no confirmed TIP cases reported in Burkina Faso and Madagascar. Overall, most investigated cases (575/608 [95%]) were classified as either probable TIP (192/608 [32%]) or clinical IP (383/608 [63%]), highlighting the importance of a multilayered diagnostic approach to confirming and treating TIP.

**Figure 2.**
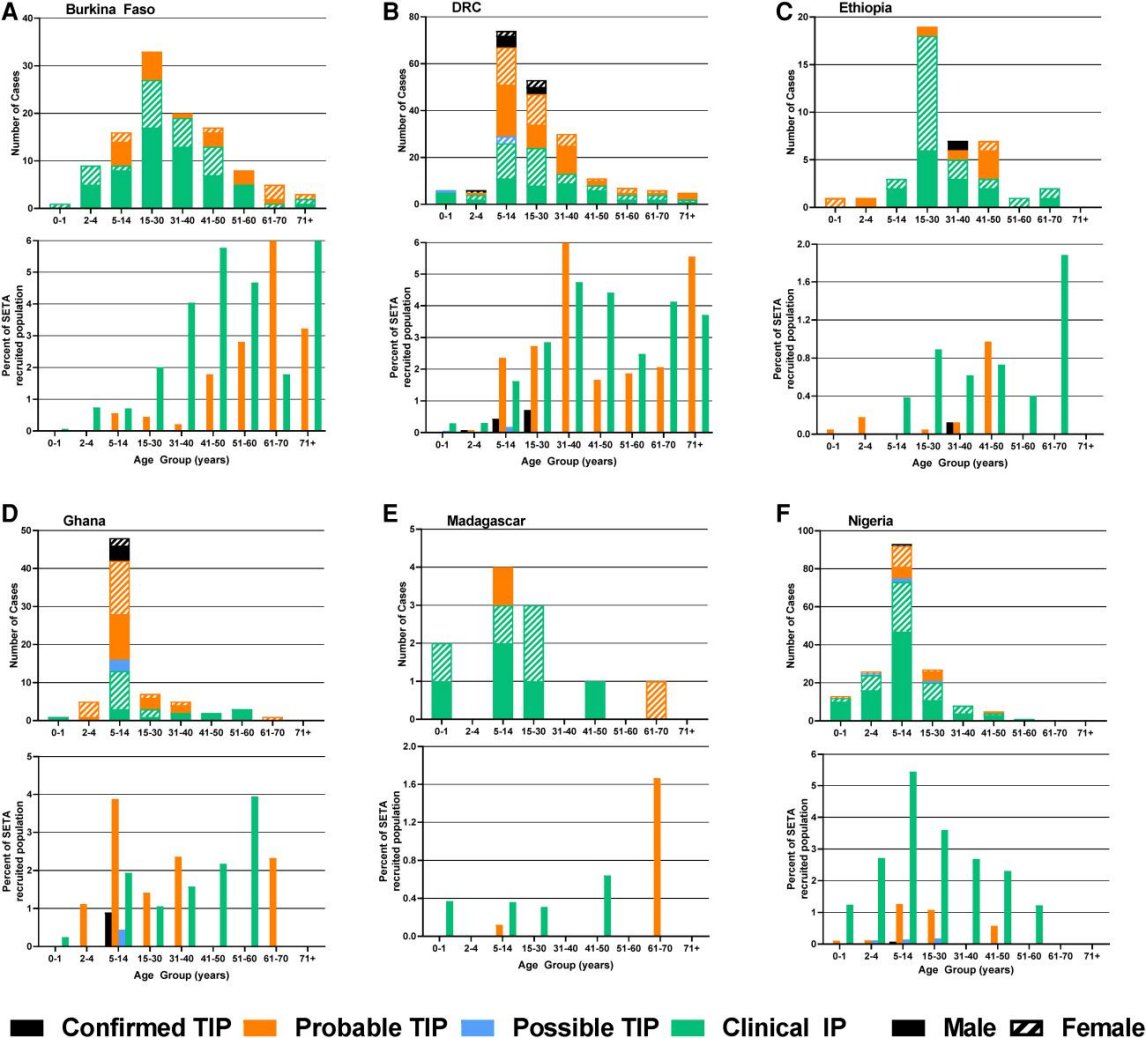
Number of cases by age and sex. *A–F*, For each country, top panel shows the number of cases and bottom panel shows the percentage of cases among the Severe Typhoid in Africa Program (SETA)–recruited population for each age group.

When evaluated by age group, as a proportion of cases evaluated in each country, TIP was highest in 5- to 14-year-olds in DRC, Ghana, and Nigeria. In Burkina Faso and Ethiopia, TIP was highest in 15- to 30-year-olds (Figure 2). However, trends by age group were not observed when evaluated as a proportion of the recruited population in each country. No sex-specific pattern was detected.

For confirmed and probable TIP cases, >90% of patients presented with abdominal pain, with 91% and 71%, respectively, having rebound tenderness on examination. More than 60% of confirmed and probable TIP cases had vomiting, and >50% had fever (Table 2). For those with possible TIP or clinical IP, >60% in each category reported abdominal pain and 55% and 41% presented with rebound tenderness in possible TIP and clinical IP cases, respectively. Abdominal distention was reported in <10% of cases across all 4 categories.

**Table 2.** Clinical Signs and Symptoms at Presentation by Category

Signs and Symptoms	Case Definition				
	Confirmed TIP (n = 22)	Probable TIP (n = 192)	Possible TIP (n = 11)	Clinical IP (n = 383)	Total (N = 608)
Signs					
ȃFever	15 (68)	103 (54)	6 (55)	229 (60)	353 (58)
ȃAbdominal pain (current)	22 (100)	180 (94)	7 (64)	258 (67)	467 (77)
ȃAbdominal distension	2 (9)	17 (9)	1 (9)	20 (5)	40 (7)
ȃJaundice	6 (27)	27 (14)	4 (36)	42 (11)	79 (13)
ȃRebound tenderness	20 (91)	136 (71)	6 (55)	158 (41)	320 (53)
Symptoms					
ȃShortness of breath	4 (18)	53 (28)	2 (18)	79 (21)	138 (23)
ȃAbdominal pain (history)	22 (100)	185 (96)	8 (73)	276 (72)	491 (81)
ȃAnorexia	7 (32)	38 (20)	2 (18)	38 (10)	85 (14)
ȃDiarrhea	9 (41)	76 (40)	4 (36)	112 (29)	201 (33)
ȃConstipation	5 (23)	71 (37)	1 (9)	102 (27)	179 (29)
ȃVomiting	14 (64)	126 (66)	4 (36)	181 (47)	325 (54)
ȃBlood in stool	3 (14)	16 (8)	1 (9)	18 (5)	38 (6)
ȃHeadache	11 (50)	85 (44)	5 (46)	164 (43)	265 (44)

Data are presented as No. (column %).

Abbreviations: IP, intestinal perforation; TIP, typhoid intestinal perforation.

**Figure 3.**
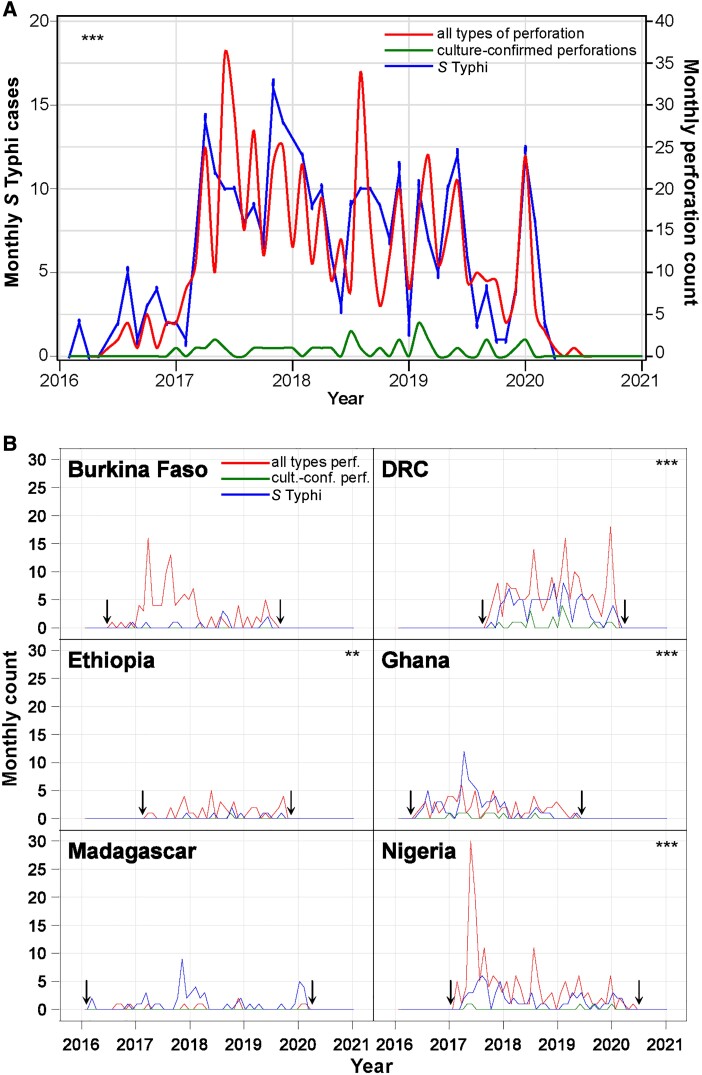
*A*, Monthly counts of typhoid culture–confirmed and all intestinal perforations (IPs) (confirmed typhoid intestinal perforation [TIP], probable TIP, possible TIP, clinical IP) from all 6 countries from February 2016 to May 2020. The monthly counts of *Salmonella* Typhi are predictive of monthly IPs. ***P < .001. *B*, Monthly *S.* Typhi and IPs by country. Arrows indicate the beginning and the end of the surveillance period and the asterisks indicate countries where monthly case counts of *S.* Typhi are predictive of monthly IPs. **P < .01, ***P < .001. Abbreviation: DRC, Democratic Republic of Congo.

Finally, to assess a seasonal correlation between IPs and overall typhoid case load, we compared the time trends of investigated IP (n = 608) as well as blood or surgical site culture–confirmed TIP to the time trends of all blood culture–confirmed typhoid cases in the SETA study. Using aggregated data for the 6 countries, the GLM analysis indicated that monthly case counts of *S.* Typhi are predictive of monthly IPs (Figure 3*A* , *P* < .001). The model estimates that for every blood or surgical site culture–confirmed case of typhoid, 1.7 (95% confidence interval, 1.4–2.0) perforations occur, approximately 1 perforation for every 0.6 culture-confirmed cases of typhoid fever presenting during the SETA study.

When the same analysis was performed for countries separately (Figure 3*B* ), a positive correlation between monthly typhoid cases and perforations was observed for DRC, Ghana, Nigeria, and Ethiopia. This association was not observed for Burkina Faso and Madagascar, perhaps due to the low number of perforations and *S.* Typhi cases overall and absence of confirmed TIP in these 2 countries.

## DISCUSSION

In this large case series embedded within a broader typhoid surveillance effort, we describe the demographic, clinical, and surgical characteristics of TIP in 6 culturally and geographically distinct countries in sub-Saharan Africa. Most cases occurred in persons aged <30 years, and in 3 of the countries (DRC, Ghana, and Nigeria), TIP peaked in the age group 5–14 years. We demonstrate that culture confirmation is uncommon, a finding consistent with TIP being a late manifestation of typhoid illness. Despite lack of culture confirmation, more than one-third of cases showed classic operative findings for TIP, and the seasonality of TIP cases correlated with the timing of typhoid detections overall. These findings are important and should be considered when determining the burden of typhoid fever and TIP in remote areas that do not have the infrastructure needed to perform bacterial culture diagnostics and pathology of surgical specimens.

Of the 608 patients investigated for nontraumatic IP, approximately one-third had TIP confirmed based on operative findings whereas two-thirds either did not have operative findings consistent with TIP or had inadequate information provided. Of the 214 surgically-confirmed cases, 22 had positive cultures for *S.* Typhi (22/214 [10%]), a ratio higher than expected based on prior reports. Of the 394 cases without surgical confirmation, 11 (3%) had positive cultures. This latter category is more consistent with prior studies in sub-Saharan Africa where culture data have low rates of positivity or where culture is not available [15]. Poor culture sensitivity has been attributed to both the delayed presentation of patients with TIP, who generally become symptomatic 2–3 weeks after initial symptoms of typhoid fever, and prior antibiotic use [16].

While TIP is a surgical emergency with high mortality, the clinical presentation is nonspecific. Patients with TIP generally presented with abdominal pain, fever, rebound tenderness, and vomiting. Current abdominal pain was the most prevalent symptom, occurring in >75% of patients in all categories combined, which is consistent with previous studies [17, 18]. Patients with surgically-confirmed (confirmed and probable cases) TIP also had higher rates of rebound tenderness and gastrointestinal symptoms including diarrhea, constipation, and vomiting, as would be expected in a patient with a perforated viscus and peritoneal inflammation.

Typhoid fever is a cyclical disease that varies based on rainfall, temperature, and region [19]. Investigated cases of nontraumatic IP followed similar trends to total overall typhoid cases, reaching statistical significance in the combined country analysis. The association of perforation cases with typhoid seasonality as determined by all *S.* Typhi–positive blood cultures held for 4 of the 6 countries in our analysis—DRC, Ghana, Nigeria, and Ethiopia. This is consistent with previous literature, as monthly cases of *S.* Typhi in Malawi were predictive of monthly IPs [14]. We did not have a sufficient sample size of both blood cultures and perforations to establish a statistically significant correlation in Burkina Faso and Madagascar. When further examining trends per country, DRC and Ghana reported the highest absolute number of surgically-confirmed TIP and the highest proportion relative to all TIP evaluated (>50% in both countries). This may be due to differences in regional burden of typhoid fever or in surgical documentation practices resulting in more accurate estimates of TIP cases.

Consistent with prior literature, children in this study had a disproportionately higher burden of TIP. In the DRC, Ghana, and Nigeria, the most frequently afflicted age group was 5–14 years. High-incidence regions generally had increased prevalence of TIP among children, compared to low-incidence regions, which had a broader age distribution of cases [4]. Younger age has also been found to be a risk factor for increased disease severity [20]. It is anticipated that the TIP cases in children would be vaccine-preventable if the WHO-recommended and Gavi-financed single-dose TCVs are implemented in catch-up campaigns in children through 15 years of age, followed by introduction into the routine immunization schedule.

Blood cultures for *S.* Typhi have long been the gold standard in diagnosing typhoid fever; however, many countries do not have the capabilities to process these cultures. Our study demonstrates that relying on such cultures significantly underestimates the true burden of disease. Furthermore, we show that total rates of nontraumatic IP do statistically correlate with total rates of typhoid, particularly in countries with greater childhood burden. Using TIP as a proxy for typhoid cases may allow for an easier and more straightforward method to estimate total typhoid cases, especially in low- and middle-income countries where typhoid burden is greatest.

This study has several limitations. First, there was not a standardized method for reporting operative findings; therefore, many TIP cases may have been wrongly categorized due to lack of consistency in documentation, as seen with the large number of clinical IP cases. Second, despite efforts to standardize definitions of clinical variables assessed at enrollment, we cannot exclude the possibility that significant variability existed between clinicians at different sites and in different countries since a number of the assessed variables were subjective. These biases may have contributed to differential enrollment of potential peritonitis cases. Additionally, this study was not designed to capture patients with TIP who did not present to the hospital or who did not survive to surgery. Given economic and geographical barriers to seeking healthcare at a hospital with advanced surgical services, patients may die before presenting to the hospital or, upon arrival, surgery is determined to be futile and is not offered. As a result, this study likely does not include the most severe of TIP cases seen in these areas.

A multifactorial approach has been shown to prevent typhoid fever and its complications, including TIP. This approach includes access to clean water, improved sanitation practices, better healthcare delivery systems, and the introduction of vaccines, including the WHO-recommended TCV, approved for children aged ≥6 months [1]. Several countries have introduced TCV [21, 22]; however, many countries have inadequate information to inform vaccine introduction decisions given their lack of data on disease burden secondary to poor reporting and inability to perform blood cultures. Including TIP in estimations of typhoid fever burden would allow more informed decisions on treating and preventing this serious disease.

## Supplementary Data

Supplementary materials are available at *Open Forum Infectious Diseases* online. Consisting of data provided by the authors to benefit the reader, the posted materials are not copyedited and are the sole responsibility of the authors, so questions or comments should be addressed to the corresponding author.

## Supplementary Material

ofad138_Supplementary_Data

## Notes

***Ethical approval*.** The study received ethical approval by the relevant entities within the countries that participated as well as by the International Vaccine Institutes' Institutional Review Board.

***Patient consent*.** All study participants provided written informed consent prior to inclusion in the study.

***Financial support.*** This research was funded by the Bill & Melinda Gates Foundation (grant number OPP1127988).

***Supplement sponsorship.*** This article appears as part of the supplement “Charting the Course to Meet the Challenges Ahead: Research and Developments on Typhoid and Other Invasive Salmonelloses” sponsored by the Coalition against Typhoid Secretariat, housed at the Sabin Vaccine Institute in Washington, DC and made possible by a grant from the Bill & Melinda Gates Foundation.
