# Correction to: Characterization of Typhoid Intestinal Perforation in Africa: Results From the Severe Typhoid Fever Surveillance in Africa Program

**DOI:** 10.1093/ofid/ofae256

**Published:** 2024-05-23

This is a correction to: Megan Birkhold, Shrimati Datta, Gi Deok Pak, Justin Im, Olakayode O Ogundoyin, Dare I Olulana, Taiwo A Lawal, Oludolapo O Afuwape, Aderemi Kehinde, Marie-France Phoba, Gaëlle Nkoji, Abraham Aseffa, Mekonnen Teferi, Biruk Yeshitela, Oluwafemi Popoola, Michael Owusu, Lady Rosny Wandji Nana, Enoch G Cakpo, Moussa Ouedraogo, Edgar Ouangre, Isso Ouedraogo, Anne-Sophie Heroes, Jan Jacobs, Ondari D Mogeni, Andrea Haselbeck, Leah Sukri, Kathleen M Neuzil, Octavie Lunguya Metila, Ellis Owusu-Dabo, Yaw Adu-Sarkodie, Abdramane Soura Bassiahi, Raphaël Rakotozandrindrainy, Iruka N Okeke, Raphaël M Zellweger, Florian Marks, Characterization of Typhoid Intestinal Perforation in Africa: Results From the Severe Typhoid Fever Surveillance in Africa Program, Open Forum Infectious Diseases, Volume 10, Issue Supplement_1, May 2023, Pages S67–S73, https://doi.org/10.1093/ofid/ofad138

The originally published version of this article required correction.

Firstly, due to a coding error, for the total recruited population (Supplementary Table 1), the age in months was counted as the age in years, creating misclassification of some individuals in the lower age groups. In addition, some participants with missing age were categorized as “age 0” in the total recruited population, and in one perforation case (in Ghana).

Secondly, an older version of Figure 2 was included in the article in error, whereby two participants in Madagascar were classified in the adjacent age category, and the figure legend used an old category (“suspected” instead of “clinical”).

The article has been updated with the corrected versions of Supplementary Table 1 and Figure 2.

