# Supplementary material for: Characterization of Typhoid Intestinal Perforation in Africa: Results From the Severe Typhoid Fever Surveillance in Africa Program
Source: Open Forum Infect Dis. 2023 Jun 2;10(Suppl 1):S67–73. doi: 10.1093/ofid/ofad138 (PMC10236516; doi:10.1093/ofid/ofad138)
Supplement: ofad138_Supplementary_Data [file ofad138_supplementary_data.docx]

**Supplementary Table 1** – Percent of cases of SETA population recruited by age group

| **Burkina Faso** | **Age Group (years)** | | | | | | | | |  |  |
| --- | --- | --- | --- | --- | --- | --- | --- | --- | --- | --- | --- |
|  | 0-1 | 2-4 | 5-14 | 15-30 | 31-40 | 41-50 | 51-60 | 61-70 | 71+ | Age Missing | All ages |
| N (%) | | | | | | | | | |  |  |
| **Total recruited** | 1492  (24.0) | 1207 (19.5) | 1260  (20.3) | 1347 (21.7) | 470 (7.6) | 225  (3.6) | 107 (1.7) | 56 (0.9) | 31 (0.5) | 4  (0.06) | 6199 (100) |
| *S.* Typhi case | 2  (0.1) | 2  (0.2) | 4  (0.3) | 5  (0.4) | 0  (0) | 0  (0) | 0  (0) | 0  (0) | 0  (0) | 0  (0) | 13  (0.2) |
| **Total investigated perforations** | 1 | 9 | 16 | 33 | 20 | 17 | 8 | 5 | 3 | 0 | 112 (1.8) |
| Confirmed TIP | 0 (0) | 0 (0) | 0 (0) | 0 (0) | 0 (0) | 0 (0) | 0 (0) | 0 (0) | 0 (0) | 0 (0) | 0 (0) |
| Probable TIP | 0 (0) | 0 (0) | 7 (0.6) | 6 (0.4) | 1 (0.2) | 4 (1.8) | 3 (2.8) | 4 (7.1) | 1 (3.2) | 0 (0) | 26 (0.4) |
| Possible TIP | 0 (0) | 0 (0) | 0 (0) | 0 (0) | 0 (0) | 0 (0) | 0 (0) | 0 (0) | 0 (0) | 0 (0) | 0 (0) |
| Clinical IP | 1 (0.07) | 9 (0.7) | 9 (0.7) | 27 (2.0) | 19 (4.0) | 13 (5.8) | 5 (4.7) | 1 (1.8) | 2 (6.5) | 0 (0) | 86 (1.4) |

| **DRC** | **Age Group (years)** | | | | | | | | |  |
| --- | --- | --- | --- | --- | --- | --- | --- | --- | --- | --- |
|  | 0-1 | 2-4 | 5-14 | 15-30 | 31-40 | 41-50 | 51-60 | 61-70 | 71+ | All ages |
| N (%) | | | | | | | | | |  |
| **Total recruited** | 1709 (27.4) | 1313  (21.0) | 1608 (25.8) | 842 (13.5) | 274  (4.4) | 181  (2.9) | 161 (2.6) | 97  (1.6) | 54 (0.9) | 6239 (100) |
| *S.* Typhi case | 10 (0.6) | 15  (1.1) | 47 (2.9) | 23  (2.7) | 8  (2.9) | 1  (0.6) | 4  (2.5) | 1  (1.0) | 1 (1.9) | 110  (1.8) |
| **Total investigated perforations** | 6 | 6 | 74 | 53 | 30 | 11 | 7 | 6 | 5 | 198 (3.2) |
| Confirmed TIP | 0 (0) | 1 (0.1) | 7 (0.4) | 6 (0.7) | 0 (0) | 0 (0) | 0 (0) | 0 (0) | 0 (0) | 14 (0.2) |
| Probable TIP | 0  (0) | 1  (0.08) | 38 (2.4) | 23  (2.7) | 17  (6.2) | 3  (1.7) | 3  (1.9) | 2  (2.1) | 3 (5.6) | 90 (1.4) |
| Possible TIP | 1  (0.06) | 0 (0) | 3 (0.2) | 0 (0) | 0 (0) | 0 (0) | 0 (0) | 0 (0) | 0 (0) | 4 (0.1) |
| Clinical IP | 5  (0.3) | 4  (0.3) | 26 (1.6) | 24  (2.9) | 13  (4.7) | 8  (4.4) | 4  (2.5) | 4  (4.1) | 2 (3.7) | 90 (1.4) |

| **Ethiopia** | **Age Group (years)** | | | | | | | | |  |  |
| --- | --- | --- | --- | --- | --- | --- | --- | --- | --- | --- | --- |
|  | 0-1 | 2-4 | 5-14 | 15-30 | 31-40 | 41-50 | 51-60 | 61-70 | 71+ | Age Missing | All ages |
| N (%) | | | | | | | | | |  |  |
| **Total recruited** | 382  (7.1) | 560 (10.4) | 775  (14.4) | 2022  (37.7) | 809  (15.1) | 411  (7.7) | 248 (4.6) | 106  (2) | 43 (0.8) | 10  (0.2) | 5366 (100) |
| *S.* Typhi case | 0 (0) | 0 (0) | 5 (0.6) | 0 (0) | 1 (0.1) | 0 (0) | 1 (0.4) | 0 (0) | 0 (0) | 0 (0) | 7 (0.1) |
| **Total investigated perforations** | 1 | 1 | 3 | 19 | 7 | 7 | 1 | 2 | 0 | 0 | 41 (0.8) |
| Confirmed TIP | 0 (0) | 0 (0) | 0 (0) | 0 (0) | 1 (0.1) | 0 (0) | 0 (0) | 0 (0) | 0 (0) | 0 (0) | 1 (0.02) |
| Probable TIP | 1 (0.3) | 1 (0.2) | 0 (0) | 1 (0.05) | 1 (0.1) | 4 (1.0) | 0 (0) | 0 (0) | 0 (0) | 0 (0) | 8 (0.1) |
| Possible TIP | 0 (0) | 0 (0) | 0 (0) | 0 (0) | 0 (0) | 0 (0) | 0 (0) | 0 (0) | 0 (0) | 0 (0) | 0 (0) |
| Clinical IP | 0 (0) | 0 (0) | 3 (0.4) | 18 (0.9) | 5 (0.6) | 3 (0.7) | 1 (0.4) | 2 (1.9) | 0 (0) | 0 (0) | 32 (0.6) |

**Supplementary Table 1 (continued)** – Percent of cases of SETA population recruited by age group

| **Ghana** | **Age Group (years)** | | | | | | | | |  |  |
| --- | --- | --- | --- | --- | --- | --- | --- | --- | --- | --- | --- |
|  | 0-1 | 2-4 | 5-14 | 15-30 | 31-40 | 41-50 | 51-60 | 61-70 | 71+ | Age missing | All ages |
| N (%) | | | | | | | | | |  |  |
| **Total recruited** | 413 (18.9) | 447 (20.5) | 671  (30.8) | 283  (13.0) | 127  (5.8) | 92 (4.2) | 76  (3.5) | 43  (2) | 29 (1.3) | 1  (0.05) | 2182 (100) |
| *S.* Typhi case | 3 (0.7) | 9 (2.0) | 54 (8.0) | 8 (2.8) | 1 (0.8) | 0 (0) | 0 (0) | 0 (0) | 0 (0) | 0 (0) | 75 (3.4) |
| **Total investigated perforations** | 1 | 5 | 48 | 7 | 5 | 2 | 3 | 1 | 0 | 1 | 73 (3.3) |
| Confirmed TIP | 0 (0) | 0 (0) | 6 (0.9) | 0 (0) | 0 (0) | 0 (0) | 0 (0) | 0 (0) | 0 (0) | 0 (0) | 6 (0.3) |
| Probable TIP | 0 (0) | 5 (1.1) | 26 (3.9) | 4 (1.4) | 3 (2.4) | 0 (0) | 0 (0) | 1 (2.3) | 0 (0) | 1(100.0) | 40 (1.8) |
| Possible TIP | 0 (0) | 0 (0) | 3 (0.4) | 0 (0) | 0 (0) | 0 (0) | 0 (0) | 0 (0) | 0 (0) | 0 (0) | 3 (0.1) |
| Clinical IP | 1 (0.2) | 0 (0) | 13 (1.9) | 3 (1.1) | 2 (1.6) | 2 (2.2) | 3 (3.9) | 0 (0) | 0 (0) | 0 (0) | 24 (1.1) |

| **Madagascar** | **Age Group (years)** | | | | | | | | |  |  |
| --- | --- | --- | --- | --- | --- | --- | --- | --- | --- | --- | --- |
|  | 0-1 | 2-4 | 5-14 | 15-30 | 31-40 | 41-50 | 51-60 | 61-70 | 71+ | Age missing | All ages |
| N (%) | | | | | | | | | |  |  |
| **Total recruited** | 542 (15.6) | 492 (14.2) | 833  (24.0) | 974 (28.0) | 251 (7.2) | 156 (4.5) | 129 (3.7) | 60 (1.7) | 30  (0.9) | 6  (0,2) | 3473 (100) |
| *S.* Typhi case | 0 (0) | 1 (0.2) | 18 (2.2) | 20 (2.1) | 4 (1.6) | 8 (5.1) | 0 (0) | 1 (1.7) | 0 (0) | 0 (0) | 52 (1.5) |
| **Total investigated perforations** | 2 | 0 | 4 | 3 | 0 | 1 | 0 | 1 | 0 | 0 | 11 (0.3) |
| Confirmed TIP | 0 (0) | 0 (0) | 0 (0) | 0 (0) | 0 (0) | 0 (0) | 0 (0) | 0 (0) | 0 (0) | 0 (0) | 0 (0) |
| Probable TIP | 0 (0) | 0 (0) | 1 (0.1) | 0 (0) | 0 (0) | 0 (0) | 0 (0) | 1 (1.7) | 0 (0) | 0 (0) | 2 (0.1) |
| Possible TIP | 0 (0) | 0 (0) | 0 (0) | 0 (0) | 0 (0) | 0 (0) | 0 (0) | 0 (0) | 0 (0) | 0 (0) | 0 (0) |
| Clinical IP | 2 (0.4) | 0 (0) | 3 (0.4) | 3 (0.3) | 0 (0.0) | 1 (0.6) | 0 (0) | 0 (0) | 0 (0) | 0 (0) | 9 (0.3) |

| **Nigeria** | **Age Group (years)** | | | | | | | | |  |
| --- | --- | --- | --- | --- | --- | --- | --- | --- | --- | --- |
|  | 0-1 | 2-4 | 5-14 | 15-30 | 31-40 | 41-50 | 51-60 | 61-70 | 71+ | All ages |
| N (%) | | | | | | | | | |  |
| **Total recruited** | 963  (22.1) | 882  (20.2) | 1339  (30.7) | 556  (12.8) | 298 (6.8) | 173  (4) | 82  (1.9) | 43  (1) | 20 (0.5) | 4356 (100) |
| *S.* Typhi case | 2  (0.2) | 10  (1.1) | 47 (3.5) | 5  (0.9) | 1  (0.3) | 0  (0) | 0  (0) | 0  (0) | 0  (0) | 65  (1.5) |
| **Total investigated perforations** | 13 | 26 | 93 | 27 | 8 | 5 | 1 | 0 | 0 | 173 (4) |
| Confirmed TIP | 0 (0) | 0 (0) | 1 (0.07) | 0 (0) | 0 (0) | 0 (0) | 0 (0) | 0 (0) | 0 (0) | 1 (0.02) |
| Probable TIP | 1 (0.1) | 1 (0.1) | 17 (1.3) | 6 (1.1) | 0 (0) | 1 (0.6) | 0 (0) | 0 (0) | 0 (0) | 26 (0.6) |
| Possible TIP | 0 (0) | 1 (0.1) | 2 (0.2) | 1 (0.2) | 0 (0) | 0 (0) | 0 (0) | 0 (0) | 0 (0) | 4 (0.1) |
| Clinical IP | 12 (1.2) | 24 (2.7) | 73 (5.5) | 20 (3.6) | 8 (2.7) | 4 (2.3) | 1 (1.2) | 0 (0) | 0 (0) | 142 (3.3) |
